# Supplementary material for: Efficacy of Loop-Mediated Isothermal Amplification for H. pylori Detection as Point-of-Care Testing by Noninvasive Sampling
Source: Diagnostics (Basel). 2021 Aug 25;11(9):1538. doi: 10.3390/diagnostics11091538 (PMC8467764; doi:10.3390/diagnostics11091538)
Supplement: Supplementary file 1 [file diagnostics-11-01538-s001.zip › diagnostics-1296552-Table S1.pdf]

Table S1. Distribution of *H. pylori* Detected by LAMP and A2143G Mutation by LAMP-RFLP in an endoscopy-room based study.

Patients	Different Sources					Total Detected No.
	Saliva	Oral Brushing	Corpus	Antrum	Fecal	
1	Undetected	Undetected	Undetected	Undetected	Undetected	0
2	Undetected	Undetected	Undetected	Undetected	Undetected	0
3	Undetected	Undetected	Undetected	Undetected	Undetected	0
4	Undetected	Undetected	-	-	Detected	1
5	Undetected	Undetected	Undetected	Undetected	Undetected	0
6	Undetected	Undetected	Undetected	Undetected	Detected	1
7	Undetected	Undetected	Undetected	Undetected	Undetected	0
8	Detected	Detected	Undetected	Undetected	Detected	3
9	Undetected	Detected	Undetected	Undetected	-	1
10	Undetected	Detected	Detected	Detected	Detected	4
11	Detected	Detected	Detected	Detected	-	4
12	Detected	Detected	Detected	Detected	Detected	5
13	Detected	Undetected	Detected	Detected	Detected	4
14	Detected	Detected	Detected	Detected	Undetected	4
15	Detected	Detected	Detected	Detected	Undetected	4
16	Detected	Detected	Detected	Detected	Detected	5
17	Detected	Detected	Undetected	Detected	Detected	4
18	Detected	Detected	Detected	Detected	Detected	5
19	Undetected	Undetected	Detected	Detected	Detected	3
20	Detected	Detected	Detected	Undetected	Undetected	3
21	Undetected	Undetected	Detected	Detected	Undetected	2
22	Detected	Undetected	Undetected	Detected	Undetected	2
23	Detected	Undetected	Undetected	Detected	-	2
24	Undetected	Undetected	Undetected	Undetected	Undetected	0
25	Undetected	Undetected	Undetected	Undetected	Undetected	0
26	Undetected	Undetected	Detected	Undetected	Detected	2
27	Undetected	Undetected	Undetected	Undetected	Detected	1
28	Detected	Detected	Detected	Detected	Undetected	4
29	Detected	Detected	Undetected	Undetected	-	2
30	Undetected	Detected	Detected	Detected	Undetected	3
31	Detected	Detected	Undetected	Undetected	-	2
32	Undetected	Undetected	Detected	Detected	-	2
33	Undetected	Detected	Undetected	Undetected	Undetected	1
34	Detected	Undetected	Detected	Undetected	Undetected	2
35	Undetected	Undetected	Detected	Detected	Undetected	2
36	Undetected	Detected	Undetected	Undetected	Detected	2
37	Detected	Detected	Detected	Detected	Undetected	4
38	Undetected	Undetected	Detected	Detected	Undetected	2
39	Undetected	Undetected	Detected	Undetected	Undetected	1
40	Undetected	Undetected	Undetected	Undetected	Undetected	0
41	Detected	Detected	Detected	Detected	Undetected	4
42	Undetected	Detected	Detected	Undetected	Undetected	2
43	Undetected	Detected	Undetected	Undetected	-	1
44	Undetected	Undetected	Undetected	Undetected	Undetected	0
45	Detected	Detected	Detected	Undetected	Detected	4
46	Detected	Detected	Detected	Detected	Undetected	4

47	Detected	Detected	Detected	Detected	Undetected	4
48	Undetected	Undetected	Detected	Detected	Undetected	2
49	Undetected	Detected	Detected	Undetected	Undetected	2
50	Undetected	Detected	Undetected	Undetected	Detected	2
51	Undetected	Detected	Detected	Detected	-	3
**Total samples**	51 (20.8)	51 (20.8)	50 (20.4)	50 (20.4))	43 (17.6)	245 (100)
Detected	21 (41.2)	27 (53)	28 (56)	24 (48)	15 (34.8)	115 (47)
Undetected	30 (58.8)	24 (47)	22 (44)	26 (52)	28 (65.2)	130 (53)
**Clarithromycin Resistance ***	Susceptible	Susceptible	Susceptible	Susceptible	Susceptible	Susceptible

* A2143G Mutation
