# Supplementary material for: Efficacy of Loop-Mediated Isothermal Amplification for H. pylori Detection as Point-of-Care Testing by Noninvasive Sampling
Source: Diagnostics (Basel). 2021 Aug 25;11(9):1538. doi: 10.3390/diagnostics11091538 (PMC8467764; doi:10.3390/diagnostics11091538)
Supplement: Supplementary file 1 [file diagnostics-11-01538-s001.zip › diagnostics-1296552-Figure S1.pdf]

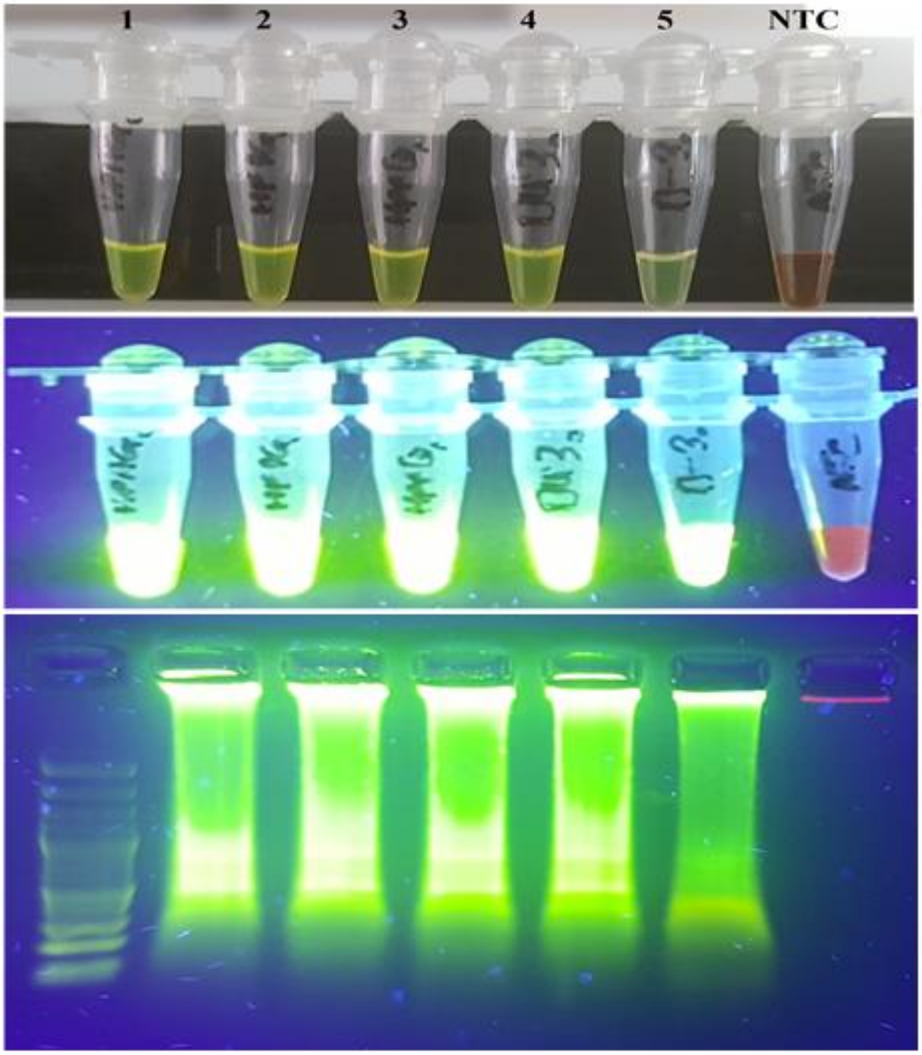

Figure S1. The LAMP assay on *H. pylori* strains (HPAG1 & DU-30) directly from colonies without any extraction as shown by SYBR Green I, naked eye, under UV and gel electrophoresis. (Ladder 100bp). Lane 1: HPAG1, Lane 2: HPAG1, Lane 3: HPAG1, Lane 4: DU-30, Lane 5: DU-30 and None-Template Control (NTC).
